# Mental and Physical Stress Responses to Personal Ultrafine Particle Exposure in Adolescents

**DOI:** 10.3390/ijerph19127509

**Published:** 2022-06-19

**Authors:** Ashley L. Turner, Cole Brokamp, Chris Wolfe, Tiina Reponen, Kelly J. Brunst, Patrick H. Ryan

**Affiliations:** 1 Division of Biostatistics and Epidemiology, Cincinnati Children’s Hospital Medical Center, Cincinnati, OH 45229, USA; cole.brokamp@cchmc.org (C.B.); wolfe.christopherlee@gmail.com (C.W.); patrick.ryan@cchmc.org (P.H.R.); 2 Department of Pediatrics, College of Medicine, University of Cincinnati, Cincinnati, OH 45221, USA; 3 Department of Environmental and Public Health Sciences, College of Medicine, University of Cincinnati, Cincinnati, OH 45221, USA; reponeta@ucmail.uc.edu (T.R.); brunstkj@ucmail.uc.edu (K.J.B.)

**Keywords:** ultrafine particles, exposure monitoring, mental health risk, adolescent mental health, adolescent emotional distress

## Abstract

Incidence rates of mental health disorders among adolescents is increasing, indicating a strong need for effective prevention efforts at a population level. The etiology of mental health disorders includes genetic, social, and environmental factors. Ultrafine particles (UFPs; particles less than 0.1 μm in diameter) have been shown to exert neurotoxic effects on the brain; however, epidemiologic evidence on the relationship between UFPs and childhood mental health outcomes is unclear. The objective of this study was to determine if exposure to UFPs was associated with symptoms of mental health in adolescents. Adolescents completed personal UFP monitoring for one week as well as a series of validated Patient-Reported Outcomes Measurement Information System (PROMIS) assessments to measure five domains of mental and physical stress symptoms. Multivariable linear regression models were used to estimate the association between PROMIS *domain T*-scores and median weekly personal UFP exposure with the inclusion of interactions to explore sex differences. We observed that median weekly UFP exposure was significantly associated with physical stress symptoms (β: 5.92 per 10-fold increase in UFPs, 95% CI [0.72, 11.13]) but no other measured domains. Further, we did not find effect modification by sex on any of the PROMIS outcomes. The results of this study indicate UFPs are associated with physical symptoms of stress response among adolescents, potentially contributing to mental health disorders in this population.

## 1. Introduction

According to the National Survey of Children’s Health, in 2016, the prevalence of anxiety, depression, and behavior or conduct problems in children aged 12–17 years was 6.1%, 10.5%, and 7.5%, respectively [1]. Additionally, the frequency of diagnosis of these disorders increases with age [1], suggesting adolescents are at an increased risk. During adolescence, major morphological and functional changes typically occur in the brain (e.g., increasing white matter volume, increase in activation of risk-taking and sensation seeking areas of the brain, increase in synaptic pruning, and developmental changes in the dopaminergic system), making this an important period of development and potentially conferring a vulnerability to certain types of psychopathologies [2]. Among adolescents aged 15–19 years, depressive disorders alone accounted for over 1200 disability-adjusted life years (DALYs) per 100,000 people in 2019, and self-harm was the second leading cause of death (9.4 per 100,000) [3], warranting the need for prevention efforts. In addition to genetic susceptibility, social factors such as socioeconomic status, ethnicity, and peer relationships play an important role in mental health during adolescence [4].

Several population studies as well as experimental models have shown that environmental pollutant exposure, including particulate matter (PM), ozone (O_3_), and nitrogen dioxide (NO_2_) during childhood and in utero, may play a role in the etiology of these disorders [5]. Epidemiological and toxicological studies have shown that, in addition to cardiopulmonary health effects, exposure to PM may also adversely impact the developing central nervous system and brain [6,7] through multiple pathways including neuroinflammation [8,9] and stimulation of the hypothalamic–pituitary–adrenal (HPA) stress axis [10]. Previous studies have identified PM can activate neuronal cell death through oxidative stress creating neurodegeneration [11] and morphological changes within the brain such as decreased white matter volume among older women of a prospective study [12], supporting the hypothesis that PM exposure is associated with adverse neurobehavioral outcomes [13,14,15]. Epidemiologic studies in school-aged children have also found significant inverse associations between air pollution exposure and decreased intelligence and memory [16] and attention deficits [17]. More recently, childhood exposure to traffic-related air pollution was found to be significantly associated with self-reported anxiety and depression at age 12 [18] and acute PM_2.5_ was related to pediatric psychiatric emergency department utilization [19].

Compared to other size fractions of PM, ultrafine particles (UFPs, particles < 0.01 μm in diameter) may be more neurotoxic due to their ability to translocate across the blood brain–barrier [20] and travel via the olfactory nerve to the brain [21], where they may exert additional neurotoxic effects such as decreased learning and memory and increased impulsivity [22]. To our knowledge, only one study has identified UFP-specific effects on mental health among an elderly population [23]. However, the relationship between UFPs and neurobehavioral effects, including the development and exacerbation of mental health disorders in children, is largely unknown. Therefore, the objective of the present study was to examine the association between adolescents’ personal exposure to UFPs and self-reported symptoms of anxiety, depression, and stress.

## 2. Materials and Methods

### 2.1. Study Population

Participants were enrolled in the Ecological Momentary Assessment of Personal Particle Exposure (EcoMAPPE) study, as previously described [24]. Briefly, between 2017 and 2019, we recruited 118 asthmatic and non-asthmatic adolescents between the ages of 13 and 17 years residing in the Cincinnati, OH region. Adolescents planning to reside in the same residence for 12 months and those who were non-smokers were eligible for enrollment. We attempted to enroll one-half of the study population to have asthma, defined as a caregiver report of a previous physician diagnosis. Caregivers provided written informed consent, and adolescents gave their assent to participate. Study protocols were approved by Cincinnati Children’s Hospital Institutional Review Board (2017-1068).

### 2.2. Ultrafine Particle Exposure Assessment

Personal UFP exposures were measured using a real-time condensation particle counter (PUFP C200, Enmont LLC, Cincinnati, OH, USA) that has been shown to maintain equivalent performance against reference CPCs (±10%, [25]). Briefly, the PUFP measures particle (sized 6 nm–3 μm) concentrations up to 2 × 10^5^ particles/cm^3^ (p/cc) at a one-second resolution. In this study, participants completed one sampling session consisting of seven consecutive days of personal UFP exposure monitoring using the PUFP. Each participant was instructed to wear the PUFP for at least three hours per day (the maximum operating time of the PUFP on full charge). Sampling occurred before and after school hours (including transit to and from the school’s premises) and at home. If participants sampled on weekends or non-school days, they were instructed to turn on the PUFP one-half hour prior to leaving their house for the day to reduce variability in times of sampling.

### 2.3. Child-Reported Mental and Physical Stress Assessments

At the end of the participant’s sampling week, a follow-up clinic visit was conducted by trained staff at CCHMC where each participant completed a series of validated Patient-Reported Outcomes Measurement Information System (PROMIS) assessments for measuring physical, mental, and social health [26,27]. Specifically, we assessed five pediatric domains: anxiety, depressive symptoms, peer relationships, psychological stress experiences, and physical stress experiences. All domains contained questions pertaining to their health from the previous seven days. For each domain, a T-score was generated with a mean of 50 and a standard deviation of 10; a higher score indicates a participant is exhibiting more symptoms related to that domain.

### 2.4. Physical Activity

Fitbit activity monitors (Fitbit, Inc., San Francisco, CA, USA) were provided to participants to record the number of steps taken per day. Participants wore the activity monitors for the duration of their sampling week. Data were retrieved from the Fitbit web API, and the average number of steps per day was calculated for each participant. Activity data were used as covariates in our models described below.

### 2.5. Statistical Analysis

UFP and T-score distributions for the sample population were characterized using summary statistics. The concentration upper limit of detection range of the PUFP is 250,000 pt/cc, therefore any readings above 250,000 p/cc were replaced with 250,000 p/cc. This resulted in truncation of 1% of the total dataset. For each participant, weekly UFP concentrations were ascertained by calculating the median of available UFP measurements recorded by the PUFP. Weekly median concentrations were log-transformed for normality. Strength and direction of associations between PROMIS domains were calculated using Spearman’s rank-order correlation.

**Figure 1:**
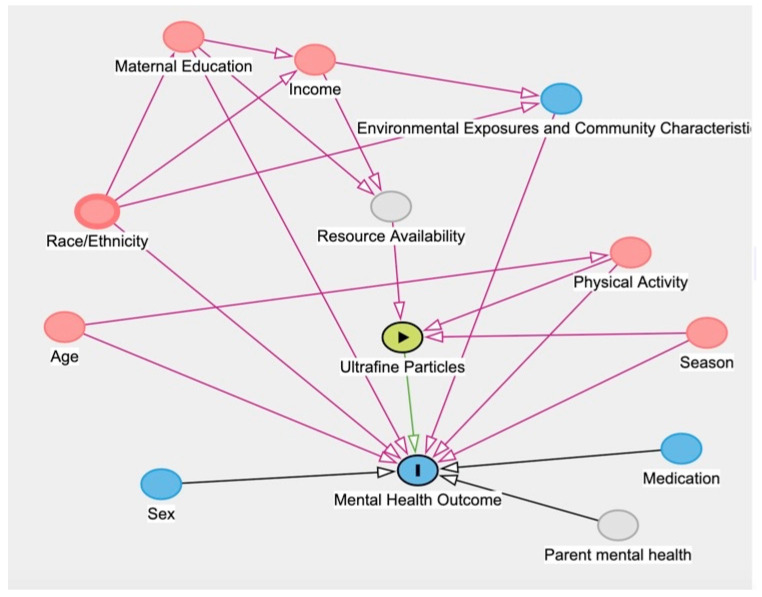
Directed Acyclic Graph (DAG) quantifying the relationship between UFPs and PROMIS *domain T* scores including confounding pathways.

We used multivariable linear regression models to assess the association between weekly median UFP exposure and child-reported mental health and stress *domain T*-scores. Individual models were built for each of the five domains (anxiety, depressive symptoms, peer relationships, psychosocial stress experiences, and physical stress experiences). In all models, we adjusted for potential confounders, identified via a directed acyclic graph (DAG; Figure 1), including the number of steps per day (collected by Fitbit), season (winter, spring, summer, or fall), maternal education (<high school, high school, and some college, or college and graduate school graduates), and household income (<$40,000 or ≥$40,000). Effect modification by sex was tested on each of the adjusted models by including an interaction term between UFP exposure and sex. Interaction terms that had a corresponding *p*-value below 0.05 were considered for further analyses in stratified models. Statistical analyses were performed using R version 3.6.1 (R Foundation for Statistical Computing, Vienna, Austria).

## 3. Results

### 3.1. Study Population

Of the 118 participants enrolled, 21 were omitted from analysis due to missing covariate data leaving a final analytical dataset sample size of 97. Of the covariates, we were missing 9 of steps per day, 12 of household income, 1 of a PUFP malfunction, and 5 of maternal education data. Participants of the analytic dataset did not differ significantly from the full dataset with respect to age, UFP exposure or PROMIS *domain T* scores. Demographics and characteristics of the sample participants used for this analysis are presented in Table 1. EcoMAPPE participants were mostly female (55.6%) and white (77.3%) with a mean age of 15.4 (±1.2) years. Participants walked on average 7060 steps a day. Most participants lived in homes with a household income greater than $40,000 (87.6%) and completed the study during summer months (36.1%). Forty-two participants (43.3%) were asthmatic; asthmatics were similar to non-asthmatics with respect to age, sex, and race. Participants with asthma reported significantly lower household income (*p* < 0.05) and lower maternal education levels (*p* < 0.01). The majority of UFP exposure measurements were recorded at participants’ homes (65%).

### 3.2. UFP Exposure

A summary of weekly UFP exposures for all participants has been described elsewhere [24]. Briefly, median UFP exposure for the entire sample ranged from 351 p/cc to 58,300 p/cc. The average mean concentration for all participants was 6792 (±7358) p/cc. Average median concentrations of UFPs were nearly twice as high during fall sampling sessions (9540 ± 10,756 p/cc) compared to summer months (5139 ± 6109 p/cc) (*p* < 0.05).

### 3.3. UFP Exposure and Mental Health Outcomes

The distribution *of T* scores for each PROMIS health domain is shown in Table 2. Anxiety scores ranged from 31.6 to 71.9 with a mean of 45.1 (SD: ±10.3) and the scores of depressive symptoms ranged from 31.7 to 76.4. Scores of physical and psychological stresses ranged from 35.2 to 77.8 and 38.4 to 71.4, respectively. Both stress experience domains had a mean score of approximately 54 (±8). In all participants, anxiety scores were moderately and positively correlated with depression symptoms (ρ: 0.72, *p* < 0.01) and psychological stress (ρ: 0.70, *p* < 0.01). Depression and psychological stress scores were also correlated (ρ: 0.73, *p* < 0.01).

**Figure 2:**
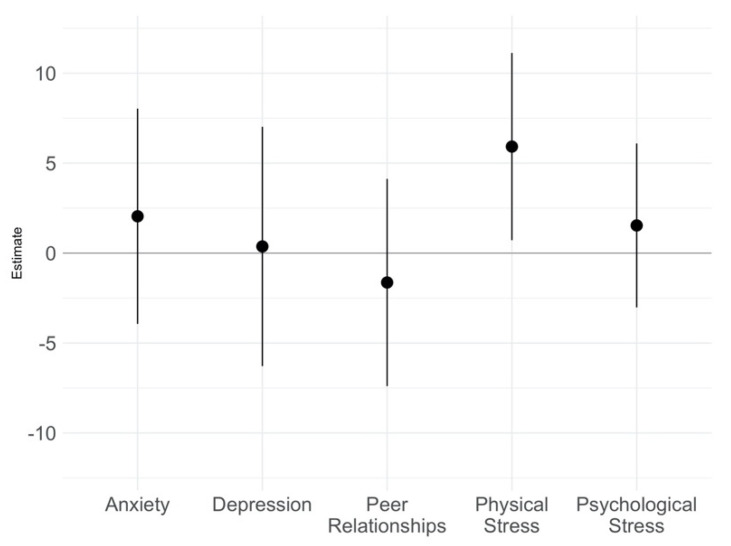
Regression coefficients and accompanying 95% confidence intervals per 10-fold increase in weekly median UFP exposure for PROMIS *domain T* scores. Models were adjusted for average number of steps per day, maternal education, household income, and season.

In adjusted models, adolescents exposed to higher weekly median UFPs reported significantly more physical stress symptoms throughout the week (adjusted β: 5.92 per 10-fold increase in UFPs, 95% CI [0.72, 11.13]) (Figure 2). UFP exposure was not associated with increased anxiety, depressive symptoms, psychosocial stress, or quality of peer relationships scores in adjusted models. When examining the interaction between UFP exposure and sex, we found no significant effect modification by sex on the relationship between increases in UFP exposure and any of the PROMIS outcomes. Therefore, models were not further stratified to determine sex-specific coefficients.

## 4. Discussion

To our knowledge, this is the first report to investigate the association between personal UFP exposure and patient-reported mental and psychological stress outcomes in a pediatric population. In our study of adolescents, increased exposure to UFPs was significantly associated with participant-reported physical stress experiences after controlling for potential confounders. However, we did not observe effect modification by sex for any of the mental health outcomes.

Only one study has examined the mental health effects of UFPs [28]. Adults over 65 years of age were assessed for depressive symptoms, and ambient UFP exposure assessment was conducted using stationary monitors. The authors observed no associations between depressive symptoms and short-term changes in UFP levels (OR: 1.04, 95% CI [0.68, 1.57]). This study differed from ours with respect to sample population characteristics, outcome assessments, and exposure sampling methods. However, our study also found no evidence of an association between depression and UFPs. Other pollutants have been associated with mental health outcomes, including PM_2.5_ and psychiatric emergency department visits (including depression) in children [19]. Outcomes described by Brokamp et al. most likely detail severe exacerbations of mental health conditions, which may explain why we did not find associations among our relatively healthy, in terms of mental health, population of adolescents. In older adults, anxiety and depression have been associated with PM_2_._5_ [29], PM_10_, NO_2_, and O_3_ [30]. Both studies used a central site sampling or spatial-temporal modeling to estimate UFPs as a proxy to individual exposure, which may have limited the results of the study. For example, in our study, the majority of sampling occurred inside the participants home, indicating individual home exposures may be more indicative of health-related effects, compared to concentrations captured outside by stationary monitors. Though we did not find similar results with respect to depression and anxiety outcomes, it is possible UFPs activate different pathways than those of PM_2_._5_ or interact directly with tissues, thereby eliciting differing symptoms of mental health [31,32]. The epidemiological literature on mental health effects of UFPs in children is lacking, therefore further research in this area is needed to confirm these relationships.

Interestingly, UFP exposure was only associated with physical symptoms of stress. While the majority of EcoMAPPE participants (80%) reported normal to mild anxiety symptom scores (*T*-score < 55), it is possible the effects on physical stress symptoms in response to UFP exposure may be a precursor to clinical anxiety symptomology or the somatization of anxiety/depression into physical distress. In fact, authors have described the physical stress domain of PROMIS as measuring “bodily manifestations of stress” [26]. Somatic symptoms, or physical symptoms of emotional distress prior to concurrent emotional disorders have been reported [33]. In the above-quoted study, out of 500 adults complaining of physical symptoms, 29% were later diagnosed with either anxiety or depression, indicating the somatization of mental health disorders potentially delays or masks these diseases leading to fewer diagnoses. Even further, one study concluded anxiety and depression were risk factors for somatic symptoms [34] while other risk factors (i.e., lower education, psychological abuse during childhood, widowed, separated, or divorced) were associated with psychiatric disorders [35]. Our findings indicated lower anxiety, depression, and psychological stress *T* scores among participants compared with physical stress scores, further confirming this possibility.

Though the central nervous system effects of UFPs is not fully understood, animal toxicological and controlled human exposure studies consistently link UFPs to several adverse events through various biological pathways including morphological changes in the hippocampus [36], learning and memory deficits [16,22], neurodegenerative changes related to Alzheimer’s disease [9], and developmental delays [37]. The two pathways proposed in the literature that may underlie these neurotoxicological effects begin with either activation of sensory nerves (HPA axis) along the respiratory tract [10] or systemic inflammation followed by neuroinflammation [9]. Studies have shown UFPs create a cascade of events in the brain leading to oxidative stress and even dopaminergic neuronal apoptosis [38] leading to increased breakdown of stress hormones [10]. In 2013, Allen and colleagues reported behavioral effects in conjunction with neuroinflammation [39]. This is especially important as it suggests UFPs activate pathways similar to stress and mental health. For example, depressive and anxiety symptoms have both been associated with increases in C-reactive protein (CRP), a marker of systemic inflammation [40]. Interestingly, myoinositol levels in the anterior cingulate cortex (a marker of neuroinflammation) have also been shown to mediate effects of traffic exposure on anxiety symptoms in children [41]. Though we did not observe significant associations of UFPs with anxiety or depression, we did find significant increases in physical stress scores, indicating the activation of the pathways described above.

We did not find significant effect modification by sex; however, females elicited greater responses to UFPs than males for anxiety, depression, and physical stress, though this result did not reach statistical significance. Sex differences of health outcomes in response to UFPs have not been widely studied in the epidemiological literature, but there is experimental evidence suggesting sex differences in hepatic toxicity [42], neurochemical and neurostructural changes [6], and neural inflammation [43]. In 2014, Allen and colleagues observed an increase in adult exposure to UFPs was significantly associated with higher norepinephrine levels, an indicator of chronic stress and anxiety, in female mice only, indicating UFP exposure can activate or perturb the HPA axis in a sex-specific manner [22].

Our study has multiple strengths including the use of personal monitors to measure individual based UFP exposures across time. In addition, the PROMIS assessments enabled us to examine symptoms experienced in the previous week that corresponded to the UFP sampling data. However, our sampling duration of three hours a day may not be an accurate representation of an individual’s true exposure patterns. The average median UFP exposure captured among participants of our study was 6792 pt/cc, regardless of the environment, which is similar to that found in several other studies on personal exposure and within schools. For example, in an exposure study among Canadian classrooms, the average indoor UFP exposure among two schools ranged from 4605 pt/cc to 5429 pt/cc [44] and in a similar study among classrooms in Texas, UFP measurements inside five various schools revealed average UFP exposures ranging from 1000 pt/cc to 5900 pt/cc [45]. Among personal exposure studies, ten volunteers in Germany recorded a median indoor UFP exposure of 7600 pt/cc to 8500 pt/cc depending on the season, similar to the median UFP exposure among EcoMAPPE participants (4340 pt/cc) [46]. Further, exposure data were collected using personal monitors in real-time, thus allowing us to determine time-based behaviors that affect UFP exposure in comparison to stationary site monitors. In a larger study with more participants and personal UFP sampling more representative of their true exposure pattern, we would expect to see a higher risk for mental health symptoms in more domains including anxiety and depression. Another limitation was that exposure to other pollutants and other size fractions of particulate matter were not measured in this study, limiting the conclusions that could be drawn on the independent effects of UFPs on mental health. However, because UFPs differ from other pollutants by their source factors, seasonal variability, and temporal distribution, and because they do not correlate well with larger particles (i.e., PM_2.5_/PM_10_) [47,48], these are unlikely to be confounders in our analysis.

In conclusion, we observed personal UFP exposure was significantly associated with increased physical stress symptom scores among adolescents. This study supports the growing public health concern for mental health risk in children and the need to identify modifiable risk factors such as air pollution exposure.

## Figures and Tables

**Table 1:** Characteristics of the study population.

Characteristic	*n* = 97
Age (years) [*n*, %]	15.4 (1.2)
Steps per Day [mean, SD]	7060 (2680)
BMI (kg/m^2^) [mean, SD]	23.3 (5.7)
Female sex [*n*, %]	54 (55.6)
Maternal Education (Some College, High School, or <High School) [*n*, %]	24 (24.7)
Household Income < $40,000 [*n*, %]	12 (12.4)
Season of Sampling [*n*, %]	
Winter	16 (16.5)
Spring	21 (21.6)
Summer	35 (36.1)
Fall	25 (25.8)
PROMIS Domain [mean, SD]	
Anxiety	45.1 (10.3)
Depressive Symptoms	45.9 (10.9)
Peer Relationships	47.9 (9.4)
Physical Stress Experience	54.0 (8.9)
Psychological Stress Experience	54.4 (7.6)

**Table 2:** Summary of PROMIS *domain T* scores for all participants.

PROMIS Domain	Mean (SD)	1st Quartile	Median (Range)	3rd Quartile
Anxiety	45.06 (10.29)	37.8	42.8 (31.6, 71.9)	51.5
Depressive Symptoms	45.93 (10.87)	37.1	43.4 (31.7, 76.4)	53.9
Peer Relationships	47.9 (9.39)	41.9	46.5 (27.2, 66.8)	53.8
Physical Stress Experience	53.98 (8.92)	48.6	53.5 (35.2, 77.8)	60.8
Psychological Stress Experience	54.42 (7.61)	49.2	53.5 (38.4, 71.4)	60.1

Values presented as *T* scores; Reference population mean of 50 and SD of 10.
